# New Appliances and Things Medical

**Published:** 1895-03-30

**Authors:** 


### NEW APPLIANCES AND THINGS MEDICAL.

[We shall be glad to receive, at our Office, 428, Strand, London, W.C., from the manufacturers, specimens of all new preparations and appliances which may be brought out from time to time.]

### "S.B.S." PURE VEGETABLE OIL SOAP.

(Stewart Brothers and Spencer, 6, Crosby Square, London, E.C.)

This new laundry soap has been for some time before the public, but the manufacturers have been carefully improving their product before delivering it in stamped tablet form. Although the various vegetable oils, of which at the present time cotton seed oil is one of the cheapest, have been used in the manufacture of soap, the "S.B.S." soap differs from many of its predecessors in being guaranteed to contain no animal fats nor resin, and thus to be the pure sodium salts of the fatty acids which occur in the vegetable oil used. From the results of our analysis we are able to confirm the statements made in connection with this soap, as we find that there is no resin present in it, and there is an entire absence of any free alkali. The total quantity of water in the sample sent us for examination was somewhat higher than that found in the two samples analysed by the firm's chemist, and amounted to 25·21 per cent. We think this amount somewhat too high, as although there is at present no definite standard as to the amount of water which should legitimately be present in a well-made soap, anything over 20 per cent. is not desirable except from the manufacturer's point of view. The high percentage of true soap in this sample gives it very good detergent properties, and the absence of any animal fats should render it free from infection, and therefore make it suitable for use for ordinary household purposes.

